# The 'diagonal' approach to Global Fund financing: a cure for the broader malaise of health systems?

**DOI:** 10.1186/1744-8603-4-6

**Published:** 2008-03-25

**Authors:** Gorik Ooms, Wim Van Damme, Brook K Baker, Paul Zeitz, Ted Schrecker

**Affiliations:** 1 Médecins Sans Frontières Belgium, Dupréstraat 94, 1090 Brussels, Belgium; 2 Department of Public Health, Institute of Tropical Medicine, Nationalestraat 155, 2000 Antwerp, Belgium; 3 Northeastern University, School of Law, 400 Huntington Avenue, Boston, MA 02115, USA; 4 Health GAP (Global Access Project), 429 West 127th Street, 2nd Floor, New York, NY 10027, USA; 5 Global AIDS Alliance, 1413 K Street NW, 4th Floor, Washington, DC 20005, USA; 6 Institute of Population Health, University of Ottawa, 1 Stewart Street, Ottawa, Ontario K1N 6N5, Canada

## Abstract

**Background:**

The potentially destructive polarisation between 'vertical' financing (aiming for disease-specific results) and 'horizontal' financing (aiming for improved health systems) of health services in developing countries has found its way to the pages of *Foreign Affairs *and the *Financial Times*. The opportunity offered by 'diagonal' financing (aiming for disease-specific results through improved health systems) seems to be obscured in this polarisation.

In April 2007, the board of the Global Fund to fight AIDS, Tuberculosis and Malaria agreed to consider comprehensive country health programmes for financing. The new International Health Partnership Plus, launched in September 2007, will help low-income countries to develop such programmes. The combination could lead the Global Fund to fight AIDS, Tuberculosis and Malaria to a much broader financing scope.

**Discussion:**

This evolution might be critical for the future of AIDS treatment in low-income countries, yet it is proposed at a time when the Global Fund to fight AIDS, Tuberculosis and Malaria is starved for resources. It might be unable to meet the needs of much broader and more expensive proposals. Furthermore, it might lose some of its exceptional features in the process: its aim for international sustainability, rather than in-country sustainability, and its capacity to circumvent spending restrictions imposed by the International Monetary Fund.

**Summary:**

The authors believe that a transformation of the Global Fund to fight AIDS, Tuberculosis and Malaria into a Global Health Fund is feasible, but only if accompanied by a substantial increase of donor commitments to the Global Fund. The transformation of the Global Fund into a 'diagonal' and ultimately perhaps 'horizontal' financing approach should happen gradually and carefully, and be accompanied by measures to safeguard its exceptional features.

## Background

The potentially destructive polarisation between 'vertical' and 'horizontal' financing of health services in developing countries has found its way to the pages of *Foreign Affairs *[1] and the *Financial Times*. [2] This debate is not new; Uplekara and Raviglione describe a pendulum that has swung between vertical and horizontal for decades. [3] However, the new International Health Partnership Plus (IHP+) gives renewed life and urgency to the debate. [4]

The opportunity offered by the 'diagonal' approach – briefly mentioned in the *Financial Times *article by Anders Nordström, Assistant Director-General of the World Health Organization (WHO) responsible for health systems and services – seems to be obscured in this polarisation. The terminology originates with Julio Frenk and Jaime Sepúlveda [5], who captured what leading AIDS activists had believed for many years: that funding for AIDS treatment and prevention will be the driving wedge for urgently needed increases in the overall level of resources available for health. Frenk and Sepúlveda describe the diagonal approach as a "strategy in which we use explicit intervention priorities to drive the required improvements into the health system, dealing with such generic issues as human resource development, financing, facility planning, drug supply, rational prescription, and quality assurance." [5]

In April 2007, the board of the Global Fund to fight AIDS, Tuberculosis and Malaria (Global Fund) agreed to consider comprehensive country health programmes for financing. [6] The IHP+ – which embraces the International Health Partnership initiated by the government of the United Kingdom [7] and related initiatives, including the Deliver Now for Women + Children campaign initiated by the government of Norway [8] – will help low-income countries to develop such comprehensive country health programmes. In a joint statement with UNAIDS, the GAVI Alliance, UNICEF, the United Nations Population Fund, the World Bank and the WHO, the Global Fund confirmed its support: "We, as international health partners committed to improving health and development outcomes in the world, welcome and fully support the International Health Partnership's mission to strengthen health systems." [7]

Similarly, discussions within the United States on the reauthorisation of the President's Emergency Plan For AIDS Relief (PEPFAR) increasingly focus on expanding human resources and improving procurement and supply chains, patient information, and laboratory systems. [9]

The authors believe that the diagonal approach is an essential concept for changing the global architecture of health assistance. This evolution could substantially broaden the scope of Global Fund financing; it might be critical for the future of AIDS treatment in low-income countries, yet it is proposed at a time when the Global Fund is starved for resources.

## Discussion

### Resource starvation and the policy preoccupations that create it

The conventional approach to health system development is that foreign assistance should make itself redundant. Sooner or later recipient countries must be able to finance health services with their own resources. Adopting this approach to the 'sustainability' of health services in low-income countries is a recipe for failure. [10] In 37 of the world's 54 low-income countries, as defined by the World Bank, public health expenditure was less than US$10 per person per year in 2004 [11] – as against the US$40 per person per year cost of an adequate package of healthcare interventions, including AIDS treatment, as defined by the Commission on Macroeconomics and Health (CMH).

The Global Fund has abandoned this conventional approach, in favour of a new form of sustainability that relies on a combination of domestic resources and predictable, open-ended foreign assistance. As the Global Fund proposal Form  for the 2007 call for proposals notes: "Applicants are not required to demonstrate financial self-sufficiency for the targeted interventions by the end of the proposal term." [12] The Global Fund's principle of 'additionality' further means that countries cannot reduce their own health spending in response to increased funding from the Global Fund.

This paradigm shift was essential, and should extend beyond priority disease programmes focused on AIDS, tuberculosis and malaria. Advocates for improved general health services should organise around this new paradigm of sustainability and additionality, and insist that donors do so as well. Donor failure on this point is one reason that general health services remain catastrophically under-funded, according to a range of observers who may agree about little else.

### The limits of the vertical approach

Buse and Waxman warned in 2001 that the vertical approach adopted by Public-Private Partnerships might create "islands of excellence in seas of under provision."[13] AIDS treatment services in low-income countries do not deserve the label 'excellence', as they often serve less than a third of the people needing treatment; they are merely islands of sufficiency. Furthermore, 'seas of under provision' sound like depths that will never be filled, while in fact it would take relatively modest resources (on a global scale) to fill them; 'swamps' might be a more appropriate image.

Thus, the metaphor of 'islands of sufficiency in a swamp of insufficiency' is useful to visualise the diagonal approach. While the vertical approach results in fragile, isolated islands of sufficiency, and while the horizontal approach leads to generalised insufficiency, the diagonal approach aims to build islands with a broad and solid base, and to gradually connect those islands, by helping to fill in the swamp, as illustrated by illustration 1 [see Additional file 1].

AIDS treatment cannot be provided in isolation from health systems. A vertical approach works for a while, and then it hits the ceiling of insufficient health workers and dysfunctional health systems, particularly in countries with high HIV prevalence. [14] Africa alone needs well over a million new health workers, [15] including 427,500 full time equivalents for universal access to AIDS treatment alone, [16] which will require expanded health education systems, in-service training systems, human resource management, skills and task shifting, and improved supervision and referral systems. Wages and working conditions must be improved across the board to retain health workers and to stop external and internal brain drains. In addition, there are growing calls for greater programme integration between priority diseases initiatives and underlying health care delivery. Because priority disease prevention and treatment requires greater coordination between health services focused on co-morbid conditions and on reaching different populations, and because priority disease programming depends ultimately on the vitality of the underlying health systems, priority disease programming must become increasing diagonal in order to be effective.

Against this background, it seems logical to argue that foreign assistance should support a diagonal approach, rather than a purely vertical or purely horizontal approach. In practice, strident advocacy for purely vertical or horizontal approaches may encourage destructive competition for resources of the kind exemplified by claims that: "HIV is receiving relatively too much money, with much of it used inefficiently and sometimes counterproductively." [17] Instead of competing, diagonal funding would follow the new realities of AIDS programming, which is becoming increasingly diagonal both in terms of integration and coordination with other disease programmes, with sexual and reproductive health, with child and maternal health, and in terms of strengthening shared health systems, e.g., labs, procurement and supply management, patient information, and human resources. In sum, diagonal funding expands resources for health system strengthening.

### How and why the IMF gets in the way

Integrating disease-specific interventions into general health services is easier said than done. Bosman describes how Zambia's tuberculosis control programme suffered immensely because of rapid integration into general health services. [18] Uplekar and Raviglione remind us that Halfdan Mahler, the WHO Director-General who was a force behind the Alma Ata goal of health for all in the year 2000, warned as early as 1966 that: "...integration is not synonymous with a laissez-faire approach. On the contrary, it requires maximum involvement of all specialized personnel such as programmers, organizers, tutors and assessors." [3] Can the resources, professionals and infrastructure of general health services of developing countries support the integration of disease-specific interventions?

Answering this question requires evidence or assumptions about whether donors are willing to commit additional resources to development assistance for health, setting aside concerns for 'sustainability,' and about whether recipient countries will in fact be permitted to use such resources. The current policies of the International Monetary Fund (IMF) present a major obstacle to expanded spending. Although the IMF's importance as a lender of last resort is declining, it must still sign off on a country's macroeconomic policies before a country is eligible for various forms of development assistance, including debt cancellation under the Multilateral Debt Relief Initiative (MDRI). The IMF's signoff is also regarded as a valuable seal of approval by foreign investors.

Although the religion of sustainability based on domestic resources has many believers, the IMF is its high priest. The IMF's assumption that development assistance is, at best, temporary and precarious and its scepticism about "fiscal expansion" have important consequences for health systems, notably in terms of ability to hire badly needed health professionals. In 2007, the IMF's own Independent Evaluation Office (IEO) confirmed the earlier observations of civil society critics that public sector wage bill ceilings were often recommended by the IMF as an element of domestic policy and that projections of the aid that recipient governments could anticipate were consistently conservative (i.e., low), leading to excessive caution with respect to what the IMF regarded as the permissible public sector wage bill or domestic primary deficit. [19]

As the domestic primary deficit is calculated as government revenue excluding grants, minus current expenditure, it is in effect a ceiling on the use of general budget support or health sector budget support. [20] De Renzio and Goldsbrough explain (about Mozambique): "Foreign-financed project lending and related expenditures were not subject to the ceiling, so the program automatically allowed for fluctuations in aid-financed project spending. However, spending financed by program aid (general budgetary support or sector-level support such as that provided to the health sector) *was *subject to the ceiling." [20] In other words: vertical financing was not subjected to the ceiling, horizontal financing – which favours channelling foreign assistance through the state budget of the recipient country (general budget support or health sector budget support) [21] – was subjected to the ceiling.

Furthermore, the IEO revealed a long-standing IMF practice that explains how health expenditure ceilings are applied without foreign assistance for health being refused. One would expect several examples of countries refusing foreign assistance, whenever donors are willing to provide more assistance than the expenditure level permitted by the IMF can accommodate. But the IMF does not prohibit countries from *receiving *more foreign assistance; it only prohibits countries from *spending *more foreign assistance. Thus, in 29 sub-Saharan countries between 1999–2005, the IMF permitted, on average, just 27 cents of every incremental dollar in foreign assistance to be used for programme expenditures, with the balance used for paying down domestic debt and accumulating foreign exchange reserves. [19] This non-spending of aid is hardly a recipe for encouraging donor confidence; indeed, it would seem calculated to perpetuate the precariousness of foreign assistance, as the IMF itself admits: "donors are reluctant to continually provide aid that is saved." [22] It also means that even if the ceilings on the public sector wage bill or on the domestic primary deficit are formally removed, the IMF can still control current horizontal financing, by requiring that aid be saved or used for debt reduction rather than spent.

In contrast, vertical financing must be used for specific purposes and it must ordinarily be added to domestic spending. Of course a recipient country might try to reduce its own contribution to the targeted intervention and to divert resources to other pressing needs. For example, in October 2002 the Ugandan finance ministry ruled that a Global Fund grant would not be allowed to lead to an increase in Uganda's health expenditure. As Bernard Rivers reported: "The Ministry said, in effect, that if the Fund provides this money, the Ugandan government will spend correspondingly less of its own money on health, leaving the health budget unchanged." [23] The Global Fund's Executive Director replied "The use of our money to save somebody else's – that's completely not allowed." [23] Although recent findings suggest that Uganda has slightly decreased its commitment to the health sector (from 9.7% of government spending in 2004–2005 to 8.3% in 2007–2008), the Global Fund at least has tried to avoid the extent to which donor funding substitutes for domestic resources. It is less clear that other sources of vertical funding have been able to accomplish this. [24]

In practice, lack of transparency about the IMF 'tax' makes direct comparisons of differences in fungibility and additionality between vertical and horizontal financing approaches difficult. Likewise, claims that vertical financing is often used inefficiently are difficult to verify and compare: if the programmes in question had not been vertical, then the funds might not have been used for their intended purpose at all.

Finally, the Global Fund's inclusion of civil society in all stages of its decision-making process, from the elaboration of proposals to watching over the implementation, strengthens its ability to ensure additionality of spending. [25] Whereas health ministries are subjected to pressure from finance ministries and the IMF, civil society would not accept those considerations as a legitimate reason for modest ambitions. As Mead Over, the former Lead Health Economist of the World Bank and presently Senior Fellow at the Center for Global Development explains (not with much enthusiasm): "By effectively converting foreign assistance from discretionary to entitlement spending, the 'success' of existing AIDS treatment programs has already locked us into a new aid paradigm." [26] Indeed, for civil society healthcare is an entitlement, a fundamental human right, not the bonus of charity or discretionary spending.

### The diagonal approach: a way into the future, at a price (worth paying)

Health GAP, the Global AIDS Alliance, and many other AIDS activists have long urged the Global Fund to support the hiring and training of an expanded health workforce, argued for broader measures of health system strengthening, and supported the integration of sexual and reproductive health and child and maternal health services with AIDS treatment. A more ambitious alternative to destructive polarisation between vertical and horizontal approaches is gradually to turn the Global Fund into a Global Health Fund, which would require that the Global Fund's resources be expanded significantly. To "consolidate towards a global health fund with one health sector funding channel" was suggested by Tore Godal, special advisor to Norway's Prime Minister as one of the options to implement the Deliver Now for Women + Children campaign, [27] and already elaborated by one of us as in terms of 'World Health Insurance'. [11]

Table 1 [see Additional file 2] illustrates both the risks and the potential rewards of a transformation of the Global Fund into a Global Health Fund aiming for public health expenditure of US$40 per person per year in 54 low-income countries in 2015. It is based on the following assumptions:

• Economies of all low-income countries will grow during 2005–2015 at the same rate as during 1995–2005;

• Population will grow as estimated by the Population Division of the Department of Economic and Social Affairs of the United Nations Secretariat, for 2015 (using the medium variant projection);

• Domestic general government revenue in 2015 will be the equivalent of 20% of Gross Domestic Product (GDP);

• Governments will allocate 15% of their revenue (or 3% of GDP) to public health expenditure.

Such a Global Health Fund would need to disburse about US$28 billion per year, assuming for purposes of argument that it did not fund any programmes in countries where per capita public health spending exceeds US$40. The CMH estimate of US$40 was calculated to cover a set of priority interventions with the infrastructure necessary to deliver them, but not the costs of training new personnel, preventive programmes like family planning, emergency care or referral hospitals. If anything, it is a conservative estimate, especially in light of new resource needs estimates for HIV/AIDS, tuberculosis, malaria, child and maternal health, sexual and reproductive health, and human resources for health and health system strengthening.

The Organisation of Economic Co-operation and Development (OECD) estimated that the combined GDP of its members was US$36,316 billion in 2006. [28] If these countries would live up to their commitment to allocate 0.7% of their GDP to foreign assistance, it would make US$254 billion. If 15% of that amount – the same percentage expected from the domestic budgets of low-income countries – were allocated to health assistance, it would make US$38 billion, in an open-ended manner. Furthermore, as we can expect the economies of the OECD to grow between 2006 and 2015, a far greater amount should be available for foreign assistance for health in 2015.

A Global Health Fund is therefore feasible, but only if donor and recipient governments are willing to abandon the conventional approach to sustainability, and only if this Global Health Fund is not subjected to IMF policies. (In theory, the latter should not be a problem, as the unpredictability of foreign assistance is the main purported justification for the IMF's conservatism about letting recipient countries spend it; foreign assistance from a Global Health Fund should be perfectly predictable.)

A Global Health Fund receiving and disbursing US$28 billion per year would require several times the annual funding level of US$6–8 billion for which the Global Fund is currently aiming. [6] The Global Fund's replenishment meeting in September 2007 resulted in a disappointing US$9.7 billion commitment for three years: a little bit more than US$3 billion per year. [29] Total annual foreign assistance for health is estimated at approximately US$12 billion in 2004, [30] and while it is possible that some of these contributions would flow instead to an expanded Global Health Fund, the proposal made here would require a *ten-fold *increase in commitments to the Global Fund. Knowing that foreign assistance for health rose from US$2 billion in 1990 to US$12 billion in 2004 [30] – a six-fold increase in total annual foreign assistance – allows for some optimism.

Such a transformation would have to go through a transitional phase of diagonal financing coupled with diagonal programming, as discussed above. Diagonal financing would help finance the disease-specific AIDS, tuberculosis, and malaria programming that is required, it would help fund increased programme integration and coordination, and it would contribute to strengthening underlying health systems.

## Conclusion

The eligibility of comprehensive country health programmes for Global Fund financing provides an opportunity and a threat. If such eligibility allows expanding health workers, increasing programme integration, and enhancing supply systems, laboratory systems, and management systems, then the Global Fund could simultaneously achieve its disease-specific and health system strengthening purposes. But if the Global Fund's diagonal intentions were undertaken without additional and sustained contributions or if a diagonal approach could not continue to bypass IMF policies, the Global Fund could be sucked into the swamp of past failed health development efforts.

Against this background, reservations are in order about IHP+. As Christopher Murray, Julio Frenk and Timothy Evans diplomatically observe: " [T]he probability that these complex efforts will have a major impact on the behaviour of donor agencies and their interactions with developing countries will be greater if they come with new resource commitments." [31] In less diplomatic words: this new global campaign looks like a rabbit-in-a-hat trick, *sans *rabbit.

## Summary

IHP+ and the Global Fund's commitment to support this new initiative(s) and their aim for comprehensive country health programmes, provides an opportunity and a threat.

The opportunity is that the Global Fund's exceptional features – its aim for international sustainability, rather than in-country sustainability; and its capacity to circumvent spending restrictions imposed by the IMF – could be extended to the improvement of health systems, and no longer be limited to disease-specific interventions. The threat is that the Global Fund might lose these exceptional features in the process of becoming a Global Health Fund.

Rather than preserving its vertical financing approach, and rather than shifting overnight to a horizontal financing approach, the Global Fund should adopt a diagonal financing approach to support increased diagonal programming. But if the Global Fund's diagonal intentions were undertaken without additional resources and without preserving long-term, sustained foreign assistance and if a diagonal approach could not continue to bypass IMF policies, the Global Fund could be sucked into the swamp of past failed health development efforts.

## Competing interests

The authors declare that they have no competing interests.

## Authors' contributions

GO developed the initial draft of this essay and contributed to later editing. He developed the tables and the illustration.

WVD reviewed and commented on successive drafts of this essay.

BKB added content on integration of priority disease programming along with simultaneous expansion of human resources for health and strengthening of vital health systems. He also helped edit the essay.

PZ proposed to expand the concept of the diagonal approach of AIDS treatment and prevention services to AIDS treatment and prevention financing, and reviewed successive drafts of this essay.

TS edited the essay, contributed to revising the policy analysis and the discussion of IMF policies, and supplied background information on current development assistance levels.

## Supplementary Material

Additional file 1 Vertical, Horizontal, and Diagonal approach. This illustration illustrates the effect of vertical, horizontal and diagonal financing approaches in countries where present government health expenditure is about US$10 per person per year. Click here for file

Additional file 2 Global Health Fund Needs. This table illustrates the needs of a Global Health Fund, aiming at increasing government health expenditure to US$40 per person per year by 2015. Click here for file

## References

[B1] Garrett L (2007). The Challenge of Global Health. Foreign Affairs.

[B2] Jack A From symptom to system. Financial Times.

[B3] Uplekar M, Raviglione M (2007). The "vertical-horizontal" debates: time for the pendulum to rest (in peace)?. Bulletin of the World Health Organization.

[B4] World Health Organization Scaling up for Better Health: IHP+ workplan of the Health 8 agencies. Report, October 2007 Geneva: World Health Organization.

[B5] Frenk J Bridging the Divide: Comprehensive Reform to Improve Health in Mexico. Lecture for WHO Commission on Social Determinants of Health, Nairobi.

[B6] Global Fund to fight AIDS, Tuberculosis and Malaria Decisions of the Fifteenth Board Meeting. Geneva.

[B7] UNAIDS International Health Partnership Launch. http://www.unaids.org/en/MediaCentre/PressMaterials/FeatureStory/20070905_feature_healtpartnership.asp.

[B8] Partnership for Maternal, Newborn and Child Health 'Orphaned' cause of women and children gains champions. http://www.who.int/pmnch/activities/delivernow/en/index1.html.

[B9] Moore A, Morrison J (2007). Health Worker Shortages Challenge PEPFAR Options for Strengthening Health Systems. Report, September 2007.

[B10] Ooms G (2006). Health Development versus Medical Relief: The Illusion versus the Irrelevance of Sustainability. PLoS Med.

[B11] Ooms G, Derderian K, Melody D (2006). Do We Need a World Health Insurance to Realise the Right to Health?. PLoS Med.

[B12] Global Fund to Fight AIDS, Tuberculosis and Malaria (2007). Proposal Form – Round 7. Geneva: Global Fund to fight AIDS, Tuberculosis and Malaria.

[B13] Buse K, Waxman A (2001). Public-Private Partneships: a Strategy for WHO. Bulletin of the World Health Organization.

[B14] Médecins sans Frontières (2007). Help wanted: confronting the health care worker crisis to expand access to HIV/AIDS treatment: MSF experience in Southern Africa.

[B15] Chen L, Evans T, Anand S, Boufford JI, Brown H, Chowdhury M, Cueto M, Dare L, Dussault G, Elzinga G, Fee E, Habte D, Hanvoravongchai P, Jacobs M, Kurowski C, Michael S, Pablos-Mendez A, Sewankambo N, Solimano G, Stilwell B, de Waal A, Wibulpolprasert S (2004). Human resources for health: overcoming the crisis. Lancet.

[B16] UNAIDS (2007). Financial Resources required to Achieve Universal Access to HIV, Prevention, Treatment, Care and Support. Geneva: UNAIDS.

[B17] England R (2007). Are we spending too much on HIV?. BMJ.

[B18] Bosman M (2000). Health sector reform and tuberculosis control: the case of Zambia. Int J Tuberc Lung Dis.

[B19] International Monetary Fund, Independent Evaluation Office (2007). The IMF and Aid to Sub-Saharan Africa.

[B20] de Renzio P, Goldsbrough D IMF Programs and Health Spending: Case Study of Mozambique. Background Paper, April 2007.

[B21] European Commission (2007). Aid Delivery Methods: Guidelines on the Programming, Design & Management of General Budget Support.

[B22] International Monetary Fund (2007). Fiscal Policy Response to Scaled-Up Aid.

[B23] Rivers B (2003). Tanzania and Uganda – Unanticipated Headaches. Global Fund Observer.

[B24] Örtendahl C The Uganda health SWAp: new approaches for a more balanced aid architecture?. Report, October 2007.

[B25] Global Fund to fight AIDS, Tuberculosis and Malaria (2007). An Evolving Partnership: The Global Fund and Civil Society in the Fight Against AIDS, Tuberculosis and Malaria. Geneva: Global Fund to fight AIDS, Tuberculosis and Malaria.

[B26] Over M AIDS Treatment as an International Entitlement: Are We Ready for a Global Welfare Paradigm?. Blog.

[B27] Godal T Global Business Plan. Presentation at the Partners' Forum of the Partnership for Maternal, Newborn and Child Health, Dar Es Salaam.

[B28] Organisation for Economic Co-operation and Development (2007). OECD in Figures 2007.

[B29] Global Fund to fight AIDS, Tuberculosis and Malaria Donors Provide US$9.7 Billion to the Global Fund. Initial Pledges for 2008–2010 Enable the Global Fund to Triple in Size. http://www.theglobalfund.org/en/media_center/press/pr_070927.asp.

[B30] Schieber G, Fleisher L, Gottret P Getting Real on Health Financing. Finance & Development.

[B31] Murray C, Frenk J, Evans T (2007). The Global Campaign for the Health MDGs: challenges, opportunities, and the imperative of shared learning. Lancet.

