# Supplementary figures and images for: The 'diagonal' approach to Global Fund financing: a cure for the broader malaise of health systems?
Source: Global Health. 2008 Mar 25;4:6. doi: 10.1186/1744-8603-4-6 (PMC2335098; doi:10.1186/1744-8603-4-6)

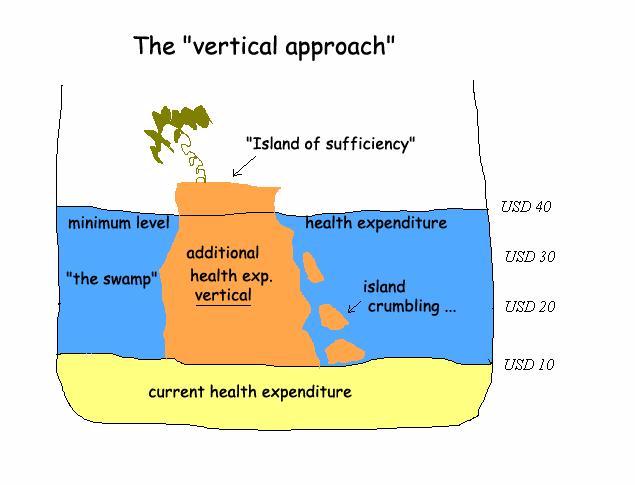


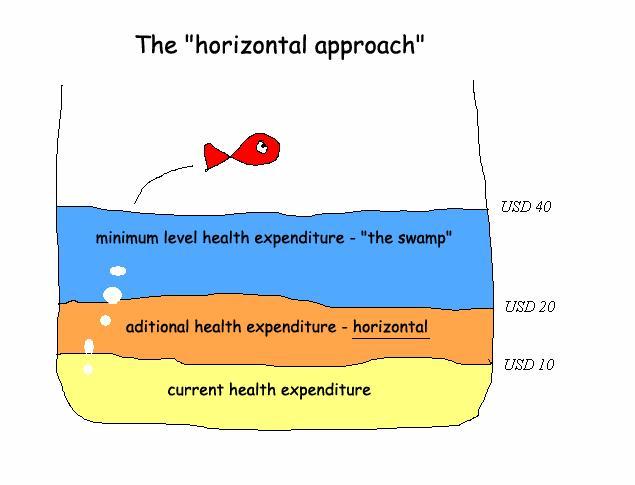


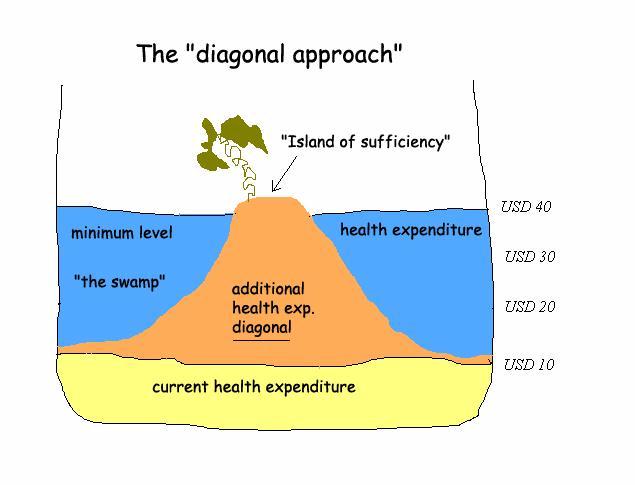


Gorik Ooms & Marc Bestgen

Supplement: Additional file 1 — Vertical, Horizontal, and Diagonal approach. This illustration illustrates the effect of vertical, horizontal and diagonal financing approaches in countries where present government health expenditure is about US$10 per person per year. [file 1744-8603-4-6-S1.doc]
